# Skeletal Findings Consistent with Signs of Rigorous Jewish Religious Practice in Four Skeletons from Qumran (Near-East), First-Century CE

**DOI:** 10.1007/s10943-024-02230-1

**Published:** 2025-01-15

**Authors:** P. Charlier, E. Conlon, I. Huynh, J. B. Humbert

**Affiliations:** 1 Laboratory Anthropology, Archaeology, Biology (LAAB), UFR of Health Sciences (UVSQ / Paris-Saclay University), 2 Avenue de la Source de la Bièvre, 78180 Montigny-Le-Bretonneux, France; 2 Department of Epidemiology and Public Health, University Hospital Raymond Poincaré (AP-HP), 104 Boulevard R. Poincaré, 92380 Garches, France; 3 Department of Radiology, Boulevard de l’hôpital, 75013 Paris, France; 4 Ecole Biblique et Archéologique Française, Couvent Saint-Etienne, 83-85 Nablus Road/Derekh Shekhem, P.O.B. 19053, Jerusalem, Israel

**Keywords:** Medical anthropology, Forensic medicine, Retrospective diagnosis, Religious practices, Paleopathology

## Abstract

Anthropological and palaeopathological examination of four male skeletons from Qumran (Near-East) revealed skeletal lesions that may be linked to an intense practice of traditional Jewish rituals within this hyper-religious community of the first-century CE: chronic inflammation of the external auditory canals linked to frequent immersion in sacred baths (*mikvah*), and osteo-articular lesions following intense and repeated genuflection and anteflexion of the trunk.

## Introduction

**Fig. 1:**
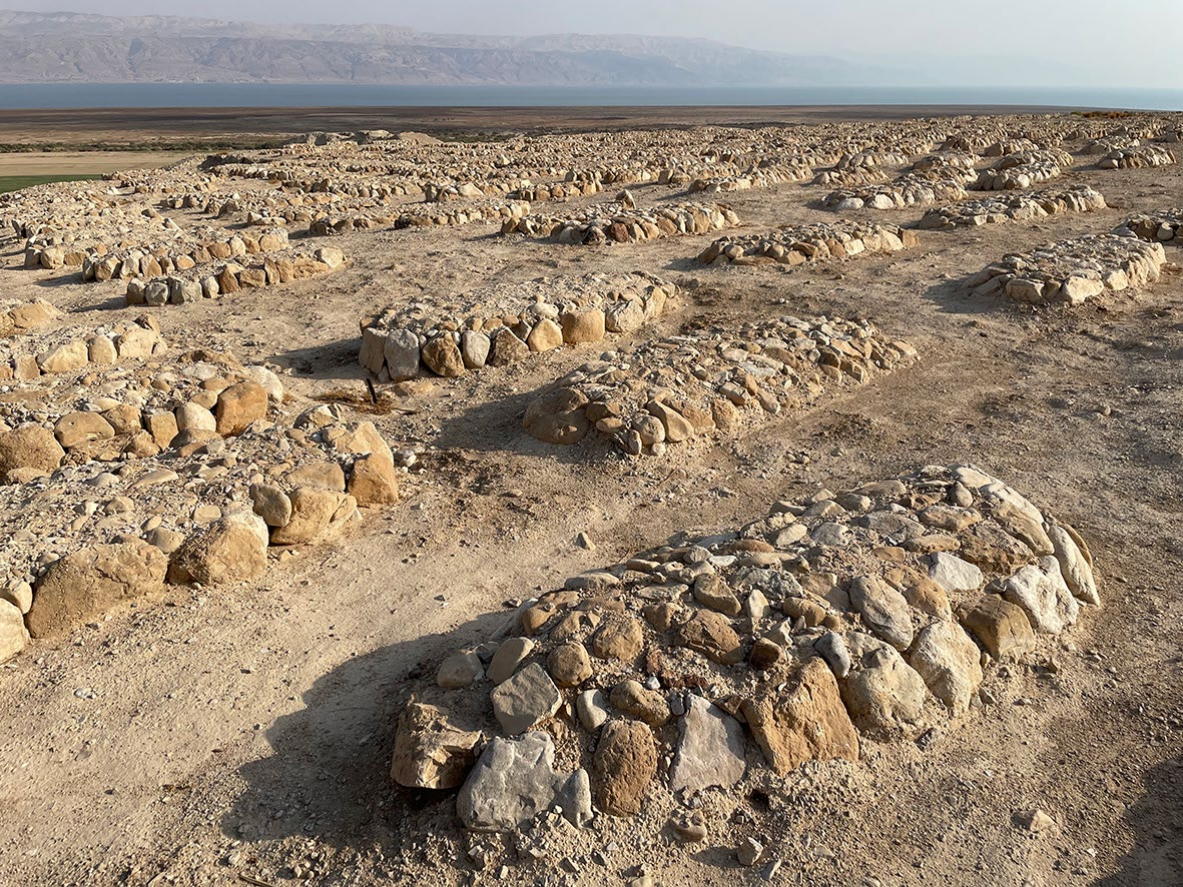
Global view of Qumran cemetery (picture P. Charlier)

Qumran is an archaeological site excavated in the 1950’s by the French Biblical and Archaeological School, associated with the Dead Sea scrolls. It is now situated in the West Bank, bordered by Jordan and the Dead Sea. According to new philological interpretations of the ancient discoveries, it is now considered a pilgrimage place and necropolis (Fig. 1) for the mystic Jewish sect of the Essenes. To date, almost fifty skeletons have been unearthed and partially studied, archaeologically identified as remains of members of the Essenes community because of a close and exclusive connection of the cemetery with the Essenian “monastery” structures (plus position of the body, absence of grave goods, position regarding the buildings, etc.) (De Vaux, 1953, 1954, 1956, 1959, 1973; Humbert & Galor, 2006).

Anthropological examinations have been carried out on the Qumran skeletons since the 1950’s, with recent medical developments (Guise Sheridan, 2002, 2003, 2006; Guise Sheridan et al., 2004). In 2023, a palaeopathological reappraisal of a sample of sixteen individuals brought to light the presence of unusual physical lesions in four of them. This article aims to show how paleopathology can highlight anatomical and pathological lesions which shed new light on historical data on the site of Qumran and its related religious practices.

## Method

The provenance of all the skeletons is the archaeological site of Qumran (Israel, first-century CE), a ruined building, isolated in the desert, occupied by the Jewish community of the Essenes, excavated by the Dominicans from the French Biblical and Archaeological School of Jerusalem since 1953. They were studied by external examination both in Jerusalem (Saint-Etienne convent) and in Paris (Musée de l’Homme) after careful cleaning and anatomical reassemblage on a table, using classical methodology (paleopathology, archaeology and forensic anthropology) (Charlier, 2008). Due to important soil deposits within and at the surface of the skeletal material, complementary analyses were performed for the skeletons from the Jerusalem site (no sampling / complementary analysis was permitted for skeletons from the Musée de l’Homme, Paris). This incorporated a radiographic and CT-scan examination carried out secondarily at Saint-Joseph Hospital, Jerusalem (Phillips 128, 156 mAs, 120 kV, slice at 0.625 mm, 28.15 mGy). All the images were independently reviewed by two MDs specialists in paleopathology, co-authors of this article (PC and IH).

## Results

A total of sixteen skeletons were examined (Table 1).

**Table 1:** Synthesis of all anthropological and palaeopathological data for the 16 studied skeletons

Skeleton (grave)	Sex	Age at death	Pathology
4	M	30–35	Metopic Suture Spina bifida occulta S3–S5 Tooth 26 abscess with alveolar bone resorption Serpens endocrania symmetrica Severe destruction of cartilage and bone at the superior/anterior part of hip articulation (coxo-femoral osteoarthritis)
5	M	30–35	Large unilateral right auditory exostosis Tooth 21 very prominent and malaligned Recent antemortem tooth 26 loss Ancient antemortem teeth 35 and 36 loss Wormian bones
T6	?	35–40	Serpens endocrania symmetrica
T7	M	40–45	Serpens endocrania symmetrica. Right coxo-femoral osteoarthritis with enthesopathies of fossa trochanterica, neck extension of head articular surface, and enthesopathies of greater trochanter
T8	M	40–45	Serpens endocrania symmetrica Unilateral right auditory exostosis Ancient antemortem teeth 25, 42 and 46 loss Abscess of teeth 12 and 15 Chronic inflammation of the palatine bone
T 10	F	45–50	Wormian bones Enthesopathies of both ischial tuberosities Agenesia for 3 molars Severe dental attrition
T11	M	Adult	Serpens endocrania symmetrica
T12	M	30–35	T syncipital (flat head—not pathological) Unilateral right mandibular condyle osteoarthritis Ancient antemortem teeth 21 and 41 loss
13	M	40–45	–
15	M	15–16	Teeth hypoplasia? Infection secondary to chronic malnutrition
T16A	M	30–40	C1/C2 severe osteoarthritis Edentulous molars Ancient antemortem teeth 21 24,28,33,35,36,37,38,41 loss Abscess in front of teeth 11 and 22/23 (with left chronic maxillary sinusitis) Ancient trauma of teeth 12 and 13 with total severance of distal end of canine Ancient tooth trauma on 15 Caries on tooth 16
T16B	M	30–40	Wormian bone Particularly prominent nuchal crest (occipital bone) Evidence of chronic gingivitis Recent antemortem tooth 37 loss Spina bifida occulta
T18	M	30–33	Bilateral acquired auditory atresia. Serpens endocrania symmetrica Cribra orbitalia Chronic pediculosis Ancient antemortem tooth 47 loss Enthesopathies on both clavicles Slight osteoarthritis on 1st thoracic vertebrae and L4 Spondylolysis of L5 Bilateral hyper-vascularisation of both anterior and posterior parts of both knees (tibia and femur)
T19	M	40–42	Wormian bones Bilateral acquired auditory atresia. Osteoma on skull vault
TA	F	Young female	3rd Trochanter on both femurs Ancient antemortem tooth 12,13,14,15 loss Abscess in front of tooth 16 Enthesopathy on tuberosity process of right radius
TB	M	> 60	Edentulous with ancient bone attrition Full ossification of thyroid cartilage Severe osteoarthritis of C1 to C5

**Fig. 2:**
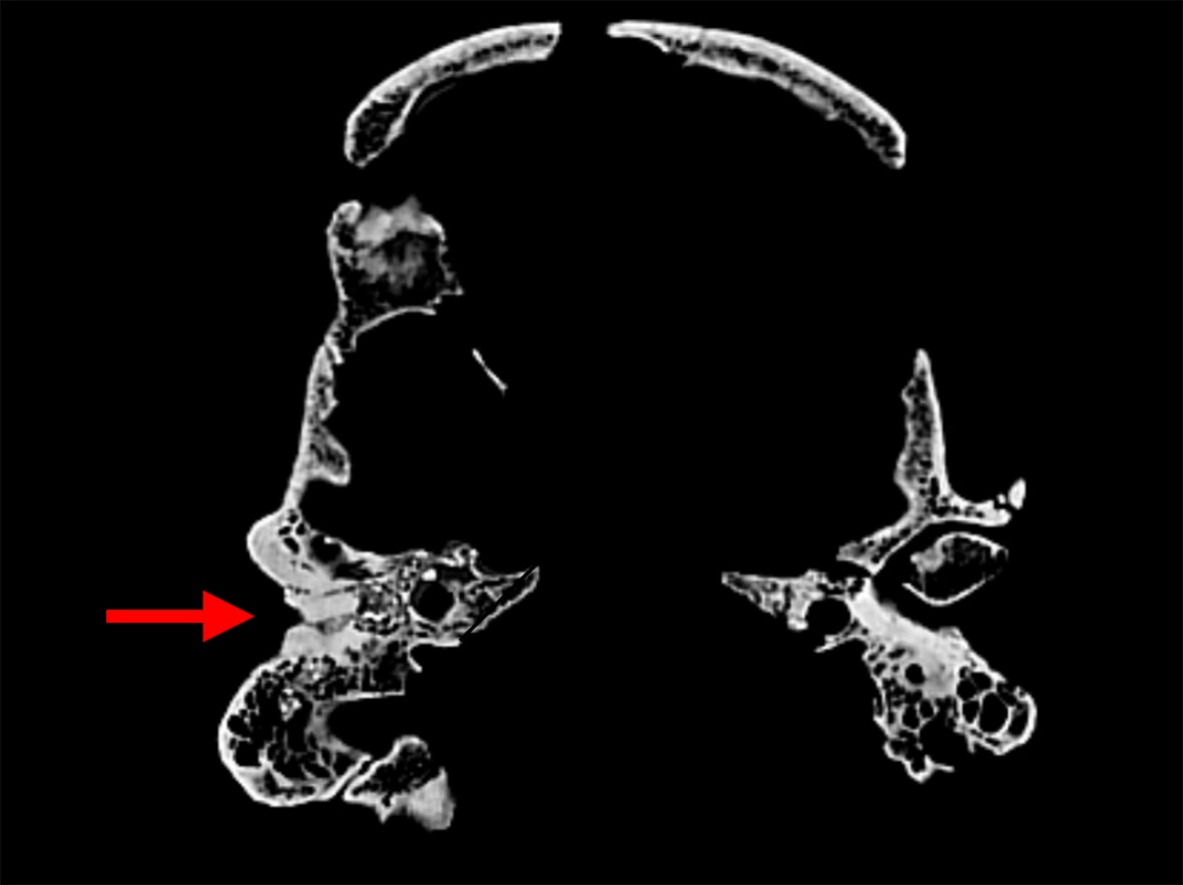
CT-scan aspect of right auricular canal exostosis of skeleton 19.2 with a partial closing of the external ear canal (red arrow / picture P. Charlier)

**Fig. 3:**
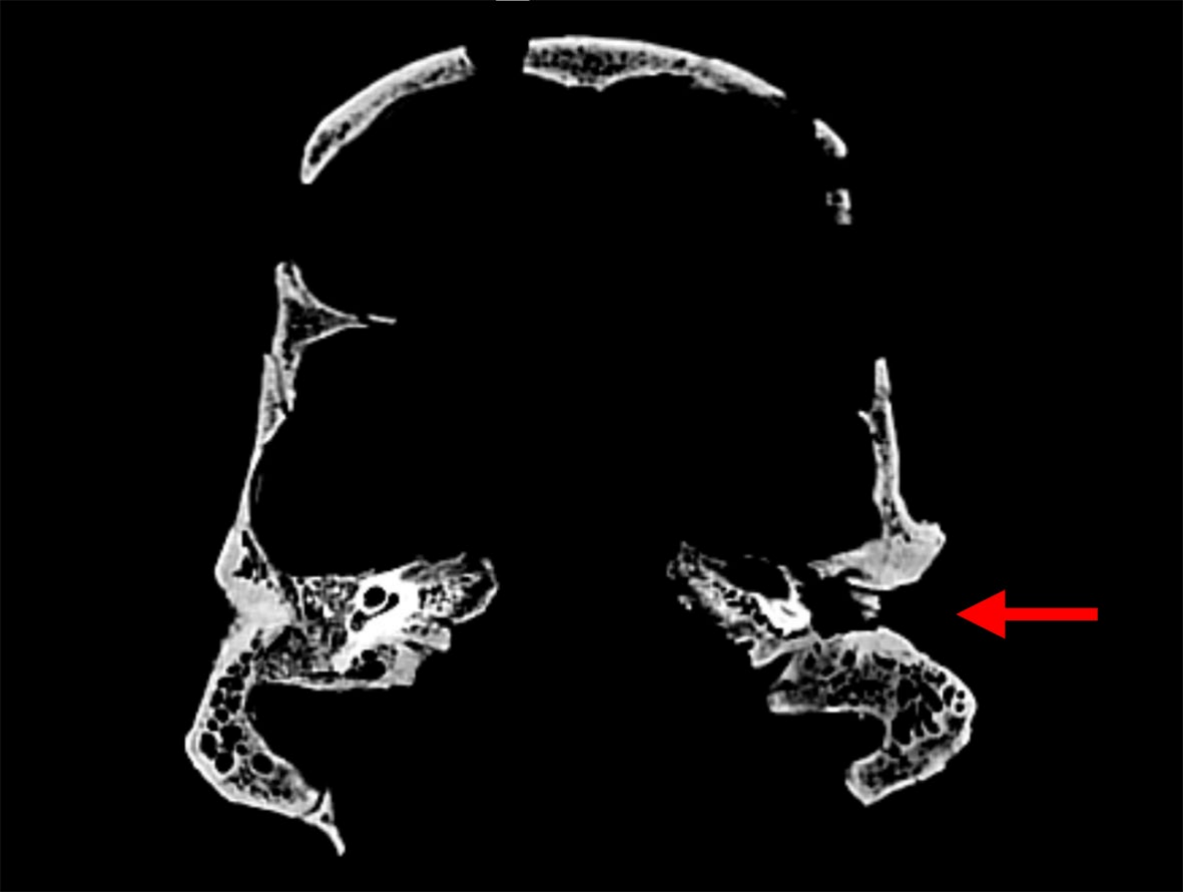
CT-scan aspect of left auricular canal exostosis of skeleton 19.2 with a partial closing of the external ear canal (red arrow / picture P. Charlier)

Tomb 19.2 contained the skeleton of a young adult male with exostoses at the level of both external auditory canals (Wegener et al., 2022). This was confirmed by CT-scan examination (Figs. 2 and 3).

**Fig. 4:**
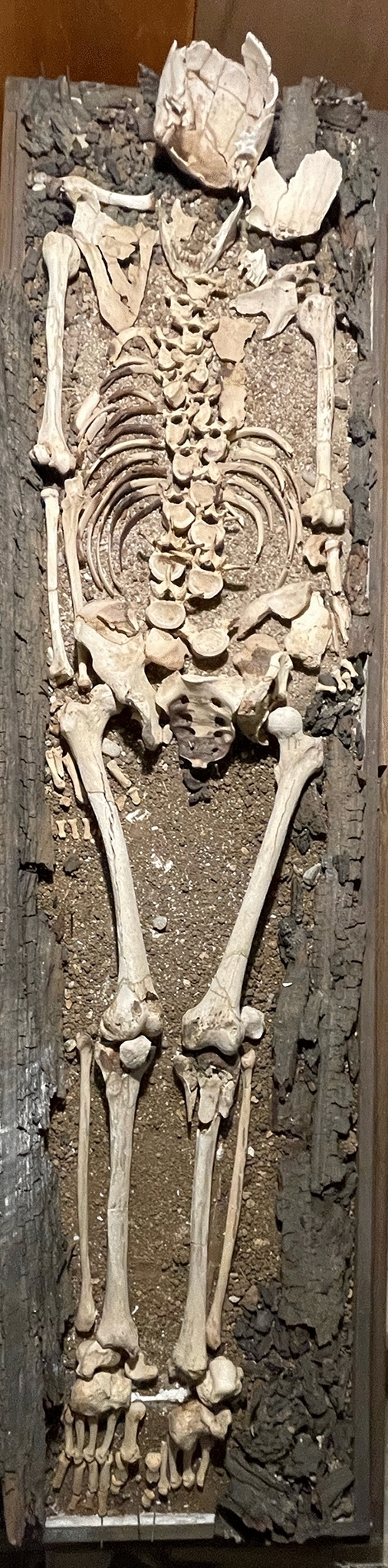
Global view of skeleton 18 (picture P. Charlier)

**Fig. 5:**
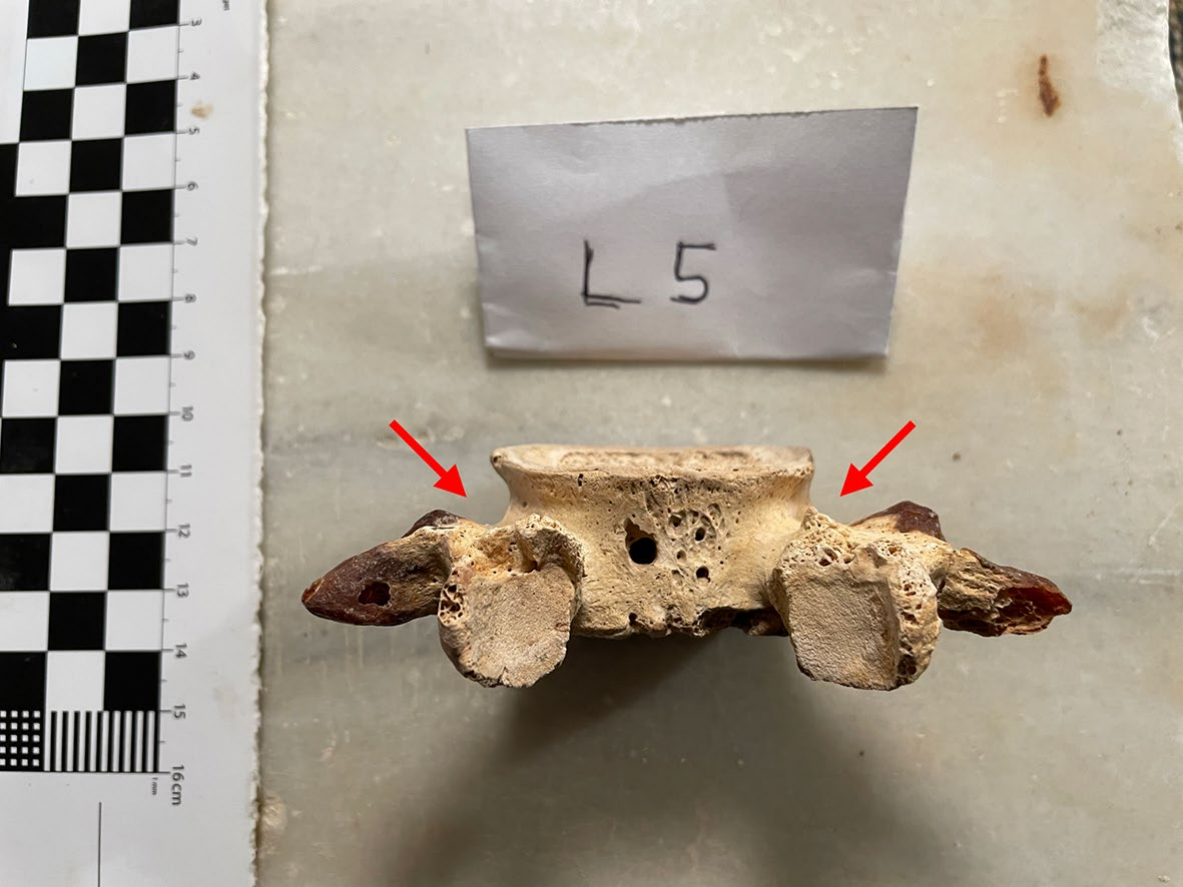
Posterior view of the fifth lumbar vertebra with acquired spondylolysis (definitive bone separation) of skeleton 18 (picture P. Charlier)

**Fig. 6:**
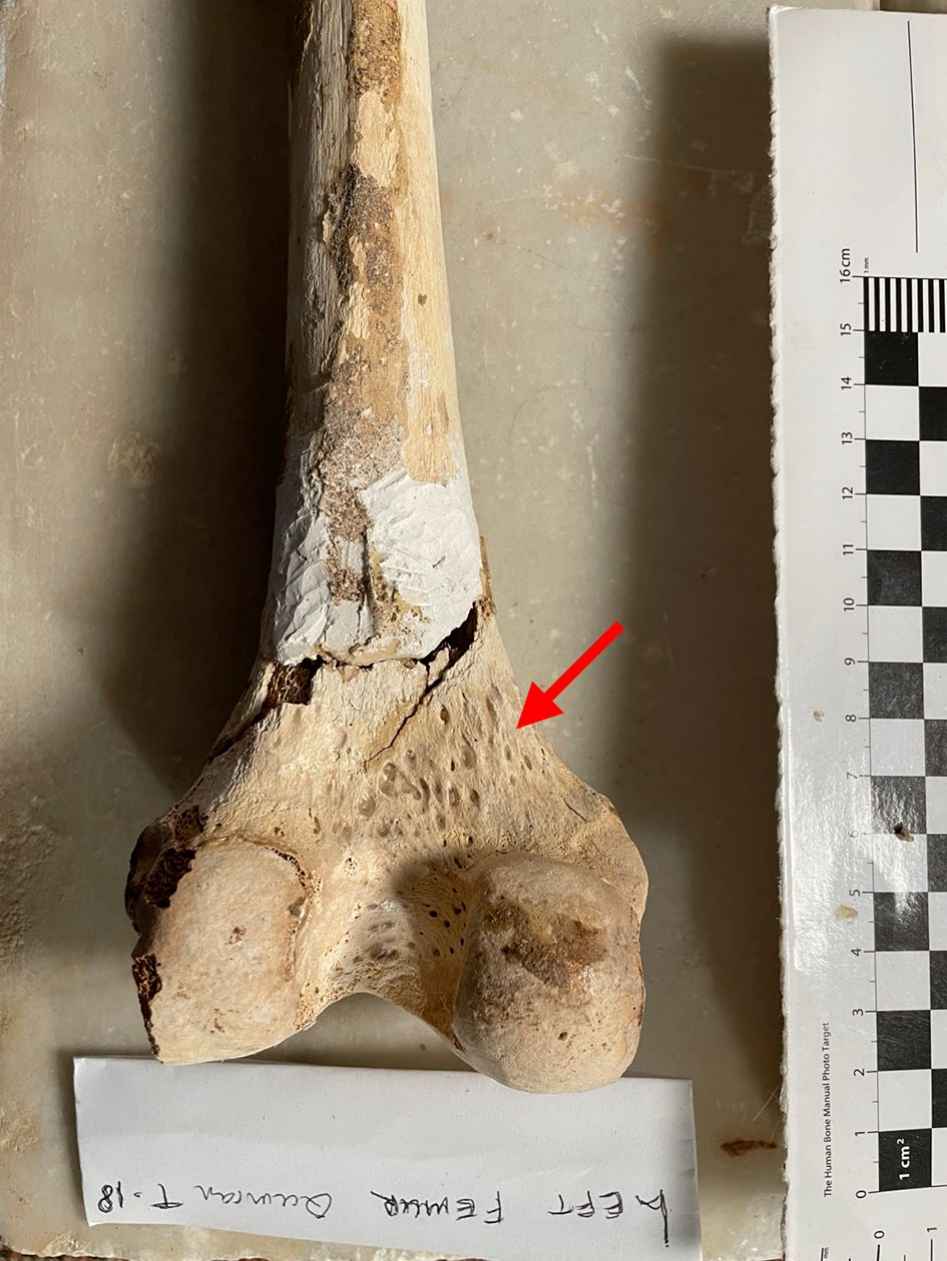
Posterior view of left femur with hyper-vascularisation (numerous millimetric holes due to chronic dilatation and high-flux of blood vessels) of the inter-condylar surface of skeleton 18 (picture P. Charlier)

**Fig. 7:**
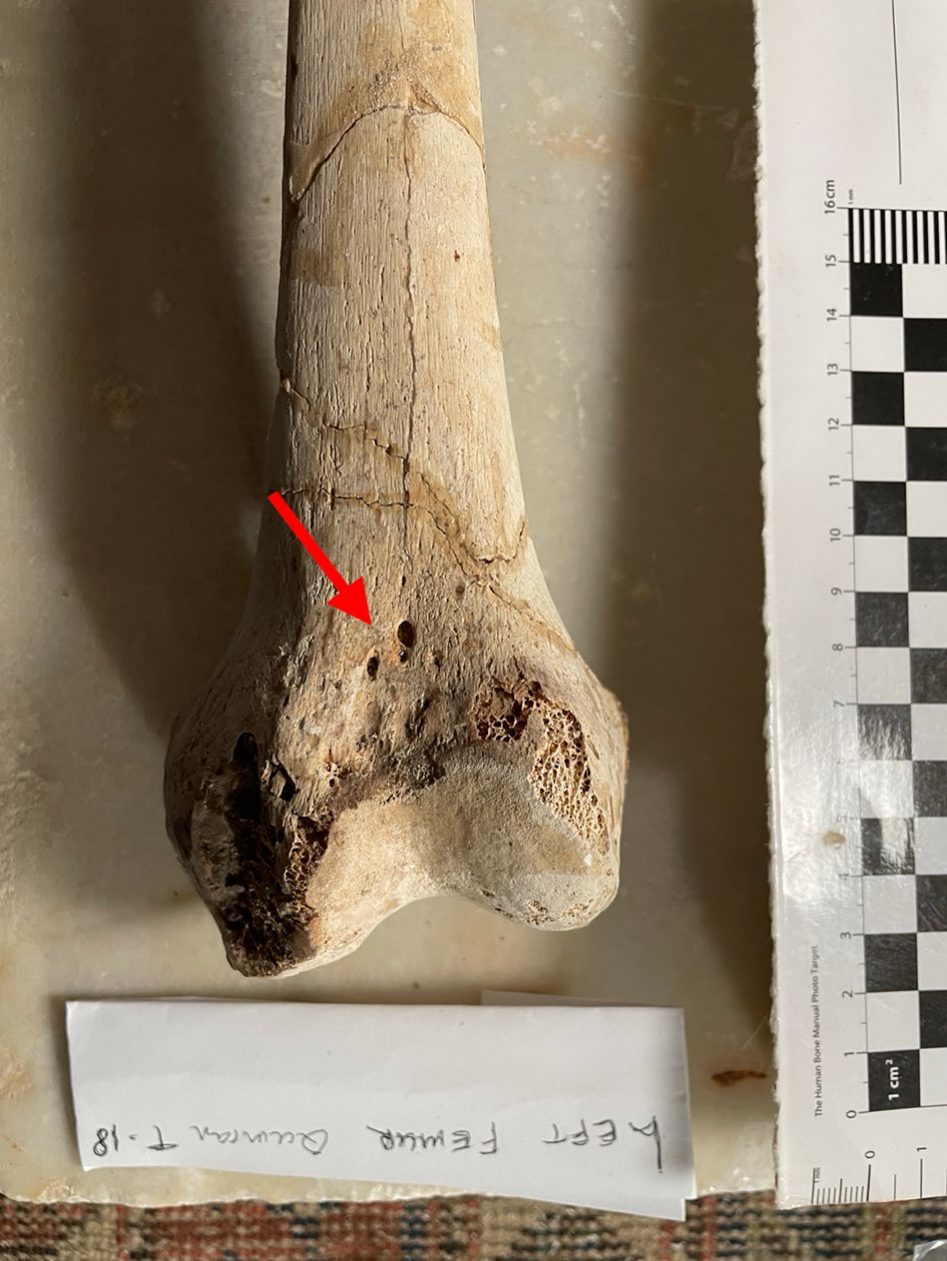
Anterior view of the left femur with hyper-vascularisation (numerous millimetric holes due to chronic dilatation and high-flux of blood vessels) of the supra-trochlear surface of skeleton 18 (picture P. Charlier)

**Fig. 8:**
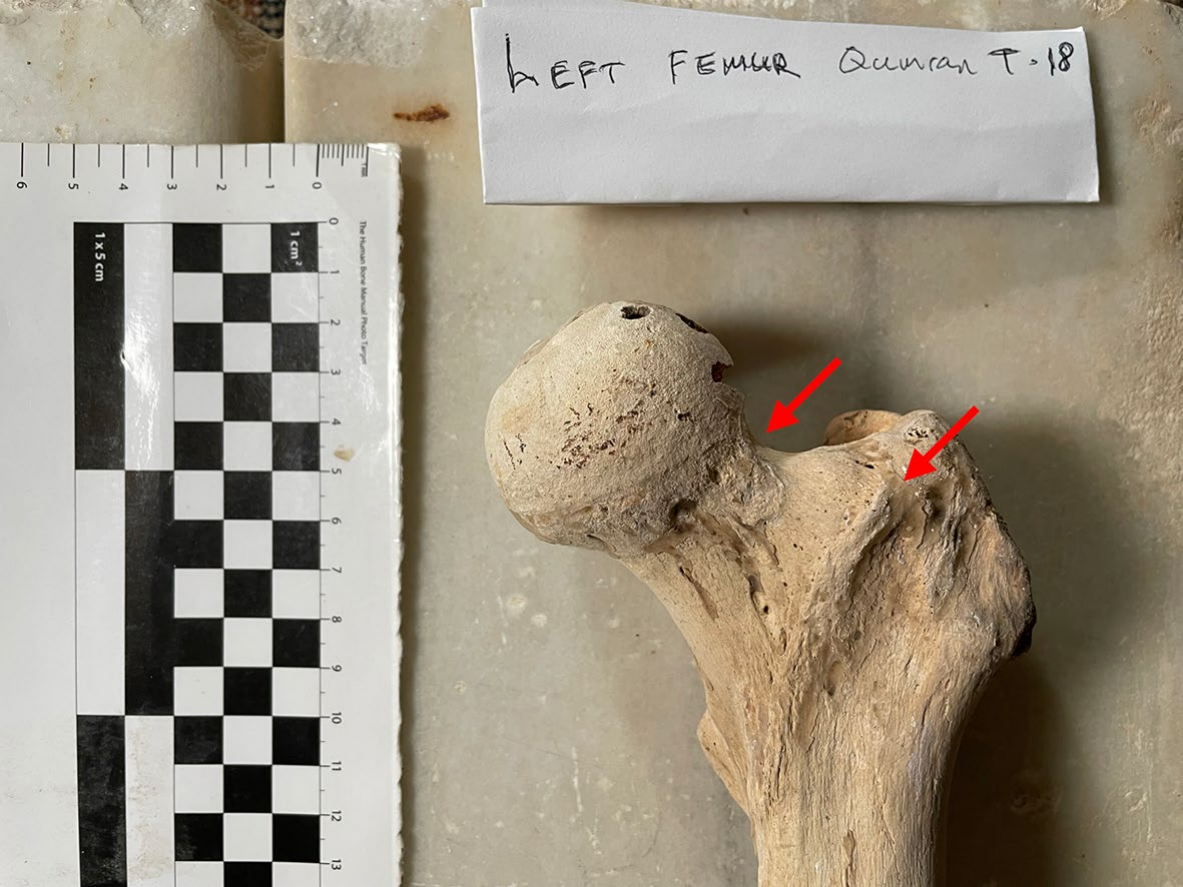
Anterior view of the left femur head, neck and great trochanter with osteo-articular modifications (Poirier facets associated with enthesopathies of the greater and lesser trochanters (i.e. *gluteus maximus* tendon insertion) of skeleton 18 (picture P. Charlier)

Tomb 18 contained the skeleton of a young adult male of 1.40 m in height. The abnormalities found were (Fig. 4):

1. Spondylolysis of the 5th lumbar vertebra (Fig. 5), due to chronic positional micro-trauma at the level of the junction between pelvis and rachis (Ottolenghi et al., 1975). Functional spondylolysis can be affirmed (and dysplastic type excluded) due to the presence of slight inflammatory remodelling on the margins of the lesion, testifying of a consequence of repeated micro-trauma, and repetitive hyperflexion / hyperextension of the trunk, leading to a definitive separation of this posterior fragment of the vertebra (vertebral pars defect) (Morita et al., 1995; Quinlan et al., 2013; Sundell et al., 2013).
2. Increase in the number and dilatation of vascular notches at the inferior part of femur and superior part of tibia, both anterior and posterior faces (Figs. 6 and 7), consistent with bilateral hyper-vascularisation of both knees, due to a hyper-use of both knees in articular and/or muscular aspects, i.e. excessive moving back and forth (multiple sinus opening from a deep infection or tumour can be excluded because of symmetrical lesions, and total absence of any other signs of infectious disease: no abscess, no fistula, no new bone formation, etc.) (Charlier, 2008).
3. Poirier facets associated with enthesopathies of the greater and lesser trochanters (i.e. *gluteus maximus* tendon insertion) on both hips (Fig. 8), possibly related to a bilateral partial “horseman syndrome” (Capasso et al., 1999).
4. Vastus notches to both patellae, i.e. an anatomical anomaly considered by some scholars a marker of activity probably due to a lack of vascularisation of this ossification zone potentially subject to the action of mechanical factors (mainly frequent squatting) (Capasso et al., 1999; Iscan & Kennedy, 1989). Other authors consider that this anomaly would be of genetic origin, having found it significantly in a population where family links were known (Angel et al., 1987; Verna et al., 2014).

**Fig. 9:**
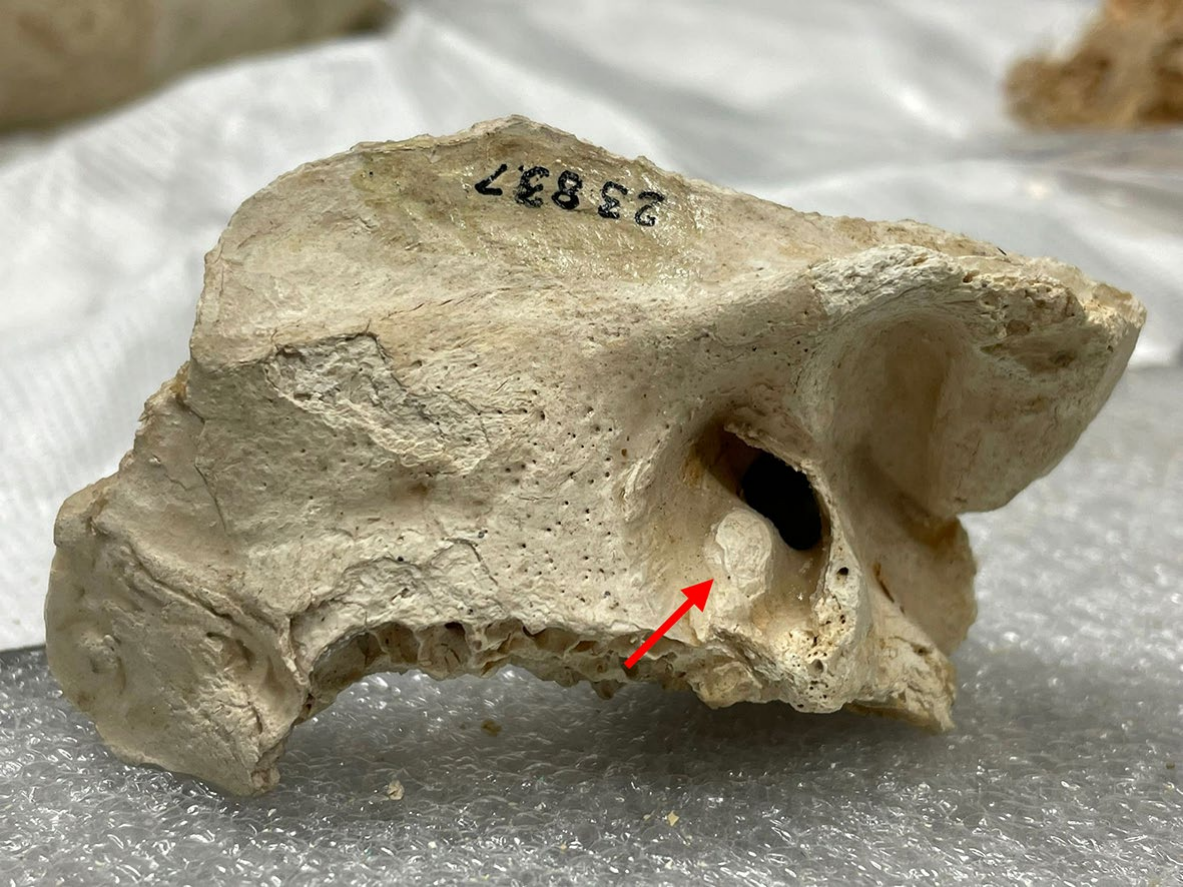
Lateral view of the right auricular canal exostosis of skeleton 8 (picture P. Charlier)

Tomb 8 contained the skeleton of a 40- to 45-year-old male who was found to have an exostosis at the level of the right external auditory canal (left side is not conserved) (Fig. 9).

**Fig. 10:**
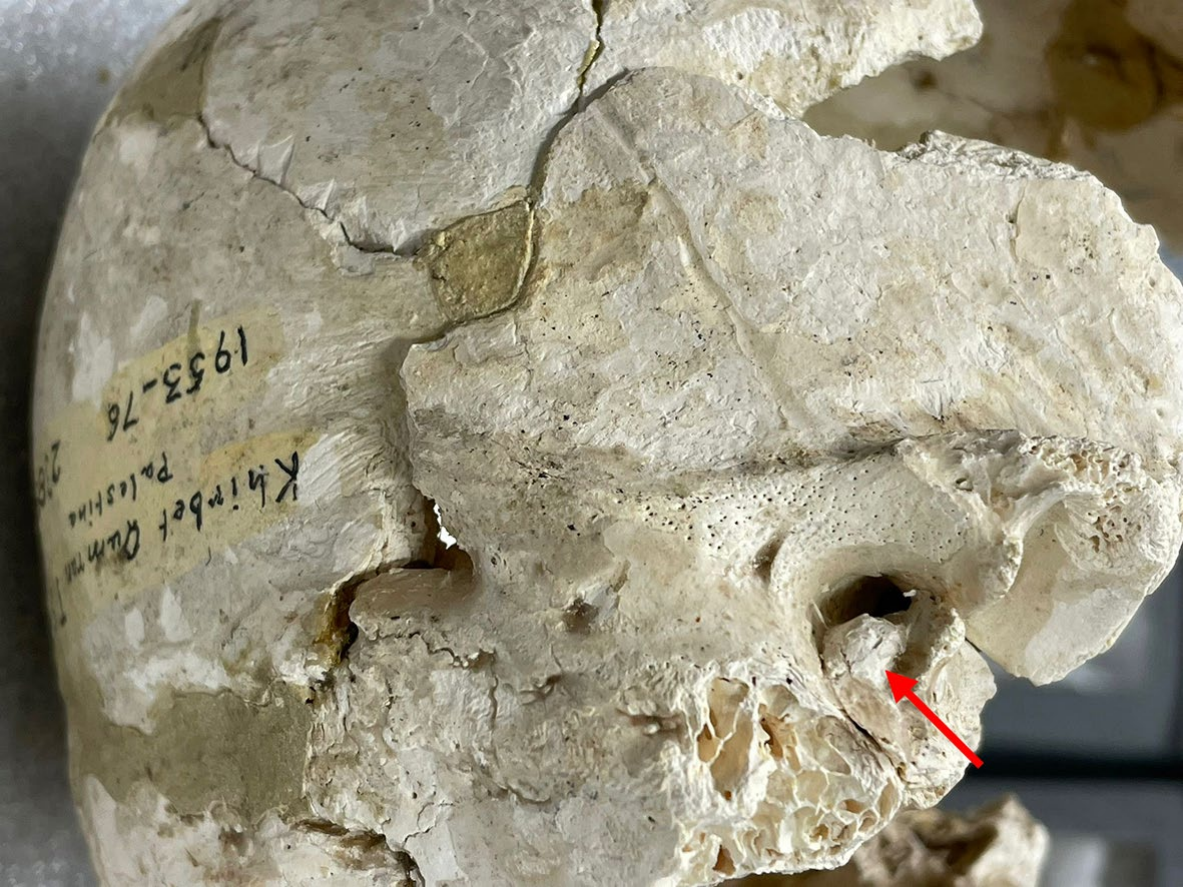
Lateral view of the right auricular canal exostosis of skeleton 5 (picture P. Charlier)

Tomb 5 contained the skeleton of a young adult male who was found to have an unilateral exostosis at the level of the right external auditory canal (left side is normal) (Fig. 10).

No cause of death was found for any of these four individuals.

## Discussion

Based upon the examination of this sample of 16 individuals, a total of four skeletons exhibited bone signs of chronic inflammation (cold exposure for external auditory canal for three of them, and strong positional and osteo-articular modifications for one of them). Such lesions were only found in male adult individuals.

The archaeological and historical context of the skeletons may provide clues to a pragmatic interpretation of such lesions. All remains are related to the Essene community, an isolated Jewish group living in the desert during the Herodian period; mentioned by Pliny the Elder (Nat. Hist. 5.17.4 [73]) (Taylor, 1999) and described at length by Flavius Josephus (*War* 4,468) (Broshi, 2007; Cornfeld, 1982) and Philo of Alexandria (*Hypothetica* 11:14–18) (Taylor, 1999). The Essenes were known as one of the three major Judaic religious groups in Herodian Judea and Roman Palestine, along with the Sadducees and Pharisees. They are documented even by ancient authors as engaging in a very ultra-orthodox form of Judaism, and critical of what they regarded as the Temple practices in “corrupted Jerusalem”, opting to strictly and rigorously follow the laws of Leviticus and Moses since the most early ages in their everyday life and routines. This, of course, involved very frequent ritual bathing and prayer (Baumgarten, 1997; Branham, 2006; Collins, 2010; Murphy-O’Connor, 1999).

Given the archaeological and historical context of these skeletal elements, a very likely hypothesis is that these lesions are, respectively, linked to frequent immersions of the head in the cold water of ritual baths (*mikvah*, such structures being particularly frequent on the site of Qumran, with more than 20 “ritual pools”) (Fig. 10) (Bohrmann, 1991), and to bodily movements of flexion of the trunk on the pelvis and thighs on the legs (i.e. repetitive bowing) during the recitation of prayers (as can still be seen in front of the Lamentation (Western) Wall, in Jerusalem).

**Fig. 11:**
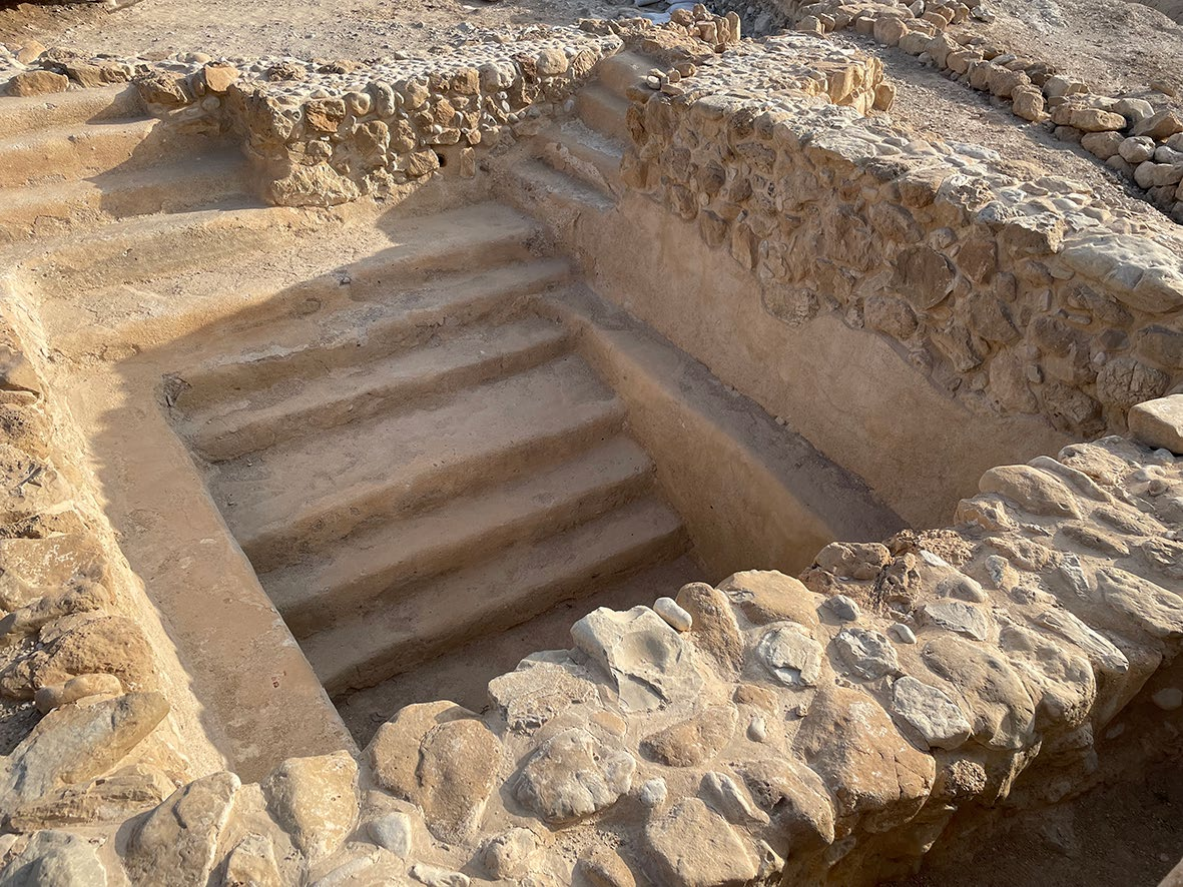
A *mikvah* from the Qumran site, dating from the period of the Essenes, previously fulfilled with pure cold water (picture P. Charlier)

Of course, other bio-medical explanations remain possible, including the practice of intense physical activities. Regarding external auditory exostoses, best known today as “surfer’s ear”, no other obvious cause of development has been reported than “repeated chronic cold water exposure leading to new bone formation at the tympanic ring” (the prevalence and severity being directly proportional to the cumulative duration and frequency of cold water exposure) (Landefeld et al., 2024), which is correlated with religious plunging for the Essenes or Orthodox Judaism (Fig. 11).

Nevertheless, the presence and concentration of such “pathological” lesions in a community known for its rigorous practice of traditional rituals are very suggestive of such a hypothesis (i.e. these lesions were likely caused by strong and intense religious activity). To our knowledge, no association of osteo-articular lesions has already been described for any ethnic group, including a Jewish sect from the past. Other examples of high occurrence rates of isolated lesions have been reported in some ethnic groups, such as spondylolisthesis (not spondylolysis, as for the Essenes) in the Inuit population from Greenland (maybe consecutive to genetic selection and chronic position of the body in the kayak) (Simper, 1986), or in the Aleut (27% known occurrence rate) and Arikara Plains Indians (9% occurrence) (Whitesides et al., 2005).

In order to resolve the genetic origin of the skeletons, indicate diets, and hint at possible disease risks and cause of death, further genetic and toxicological analyses have been carried out.

Unfortunately, DNA extraction was attempted by both the anthropologist Susan Guise Sheridan (University of Notre-Dame, Indiana, USA) and the geneticists Eva-Maria Geigl and Thierry Grangé (Jacques Monod Institute, CNRS, Paris Cité University, Paris, France) on two different samples, and proved to be a fruitless task. The collagen necessary for this was disintegrated, inadequate and beyond salvageable due to poor preservation and storage methods of the remains in the 1950s (Guise Sheridan, 2002, 2003, 2006; Guise Sheridan et al., 2004). The only possibility of extracting DNA from the skeletons would be to carry out new archaeological excavations according to modern scientific standards, with no guarantee of success given the climatological conditions of the site (perhaps the deeper burials would allow better differential conservation of ancient DNA?).

Regarding toxicological exposure of the Essene population in Qumran, two sets of analyses have been carried out previously. The first one focused on architectural samples from houses and non-domestic buildings and constructions (including water tanks and religious pools). It highlighted that the mortars and plasters contained very high levels of bromide. Some minerals were in toxic amounts and may have affected the health of the individuals who accessed water, especially to drink, from various on-site pools. However, no data were available to ascertain the degree of contamination present in the water, but Shimron recommended further analysis of the relevant human remains associated with the site. Additionally, Qumran showed high levels of lead anomaly in installation Locus 49 and a high arsenic content in installations at Loci 110 and 117 (Shimron, 2004). Other metals were tested, without any anomaly in these samples: aluminium (Al), antinomy (Sb), barium (Ba), beryllium (Be), bismuth (Bi), cadmium (Cd), gallium (Ga), germanium (Ge), gold (Au), indium (In), lithium (Li), mercury (Hg), nickel (Ni), platinum (Pt), silver (Ag), strontium (Sr), tellurium (Te), thallium (Tl), tin (Sn), titanium (Ti), vanadium (V) and uranium (U).

On the other hand, Rasmussen et al. did trace element studies on several sets of human bones (graves T12, T15, T16 × 2, T18, T19, TA, TB). The results were as follows: Qumran bone trace element levels of Zn (zinc), Ca (calcium), Sb (antimony), Co (cobalt) and Cs (cesium) of these remains were barely two standard deviations away from comparative sites from Bronze Age Denmark and Arabia. Strontium (Sr) and chromium (Cr) levels were found to be very different in the three samples, the Danish sample being the lowest, the Arabian very high, whilst Qumran strikes a happy balance between the two. Furthermore, the Qumran remains had higher than both Scandinavian and Arabian bones in bromide (Br) and selenium (Se) levels by a factor of 10 (Rasmussen et al., 2004).

All these elements, both environmental and biological, seem to have no direct consequence on the development of bone lesions described here (i.e. inflammation of the external auditive canal and diffuse osteo-articular lesions).

## Limitation

A limitation of this study is the fact that these findings are restricted to an obviously very selected population: 13 males and only 2 females (with one further of unknown sex); one juvenile, 12 young adults (30–50 yrs.) and only one over 60. Out these 16 individuals—and beyond the four aforementioned ones—several individuals show enthesopathies, arthrosis of large joints and spondylosis of cervical vertebrae. It appears likely that degenerative and/or inflammatory lesions are linked to the daily living conditions of these populations, to which may be added secondary lesions linked to ritual (religious) practices. An extension to other skeletal samples from the same archaeological site and/or the same population will be necessary to better understand the health status and functional consequences within the Essenes.

## Conclusion

To date, rigorous religious practice with frequent direct exposure to cold liquid and intense kneeing/anteflexion movements represent the best explanation for the bone lesions, after excluding all other differential diagnosis and pseudo-pathologies (i.e. post-mortem damages).

In any case, further investigations on the remaining skeletons from other anthropological collections and possibly new excavations in the future may complement our observations and emphasise the frequency of such lesions within the Essenes community, with possible severe anatomical of functional consequences (at least hearing loss, recurrent infections, otorrhea, cerumen impaction, etc. for the external auditory exostoses, and chronic axial and peripheral joint pain).
